# From scribble to scrutiny: The legal risks of poor clinical handwriting

**DOI:** 10.4102/jcmsa.v4i1.311

**Published:** 2026-04-23

**Authors:** Suhayfa Bhamjee

**Affiliations:** 1 School of Law, College of Law and Management Studies, University of KwaZulu-Natal, Pietermaritzburg, South Africa

**Keywords:** medical negligence, illegible documentation, medico-legal risk, patient records, electronic health records, documentation standards, forensic medical evidence, healthcare litigation

## Abstract

Illegible handwritten patient records pose a significant threat to clinical accountability and legal integrity in South Africa’s healthcare system. Despite clear guidelines from the Health Professions Council of South Africa (HPCSA), poor documentation remains widespread, particularly in public sector facilities. A black-letter doctrinal analysis was conducted of South African law (*Medicines and Related Substances Act* General Regulations, HPCSA Booklet 9, and the *National Health Act*) and the evidentiary treatment of clinical records in case law. A focused comparative reference to a 2025 Punjab & Haryana High Court judgement was used to contextualise the normative stakes of legibility in another common-law system. South African primary law already requires legible prescriptions and legible, understandable clinical records; courts treat hospital notes as hearsay unless properly admitted, with clarity impacting the weight accorded to such documentation. The Indian judgement constitutionally frames legibility as part of the right to health and mandates interim capital-letter prescriptions pending digitisation. Clear documentation is essential for justice, safety and the dignity of patients. The article calls for curriculum reform, digitisation, policy enforcement and legal recognition of legibility as a component of the right to access healthcare. This article may assist legal and medical professionals in recognising legibility as a constitutional obligation, thereby strengthening medico-legal accountability and promoting patient-centred care.

## Introduction

In South Africa’s medico-legal landscape, handwritten patient notes remain a common feature of clinical documentation, particularly in public health facilities.^1^ While these records are intended to serve as accurate reflections of clinical encounters, their evidentiary value is often undermined by poor handwriting, incomplete entries and inconsistent formatting. In medical negligence litigation, such documentation frequently becomes the cornerstone of both defence and prosecution arguments. Yet, when notes are illegible or inadequately recorded, they fail to meet the evidentiary threshold required in court, compromising the integrity of the legal process and the fairness of outcomes.^2,3,4^

The consequences are not merely procedural. South Africa has witnessed a marked rise in medical negligence claims over the past decade, with billions of rands paid out in damages – placing immense strain on provincial health budgets and the national fiscus. The South African Law Reform Commission has flagged this trend as a systemic risk, exacerbated by poor record-keeping and the absence of reliable clinical evidence.^5^

This article demonstrates that legibility is not a mere professional nicety: it is a legal duty in South Africa for prescriptions and a professional standard for patient records, and it materially affects the evidentiary reliability of clinical documentation. Using South African primary sources and a focused comparator from India,^6^ we distinguish what the law requires now (legibility, irrespective of medium) from what policy should pursue (progressive digitisation that meets legal standards).^4,5,7,8,9,10^

## Methodology

This article follows a black-letter doctrinal approach to South African law, analysing the *Medicines and Related Substances Act* General Regulations (Regulation 33) on prescriptions, the Health Professions Council of South Africa (HPCSA) Booklet 9^11^ on patient records, and the *National Health Act* provisions on record-keeping, alongside case law addressing the admissibility and evidentiary weight of clinical records. It then engages in targeted comparative jurisprudence by examining the Punjab & Haryana High Court’s judgement (CRM-M-30302-2024, 27 August 2025) on legible prescriptions as part of Article 21’s right to health.^5,6,7,8,9,10^

## Medico-legal risks of illegibility in South Africa

In South Africa, handwritten clinical notes and medico-legal reports (MLRs) continue to serve as primary evidence in both criminal and civil proceedings. These documents are often pivotal in cases involving sexual offences, assault and medical negligence, where the accuracy and clarity of recorded information can determine the outcome of litigation. However, when such records are illegible, incomplete or poorly structured, they undermine the evidentiary process and expose both healthcare professionals and institutions to significant legal risk.^2,11^

The HPCSA, in its Guidelines on the Keeping of Patient Records, mandates that all patient records must be ‘complete, legible and understandable’. It warns that illegible documentation may constitute unprofessional conduct and may not withstand legal scrutiny. While these standards apply irrespective of format and do not themselves mandate digitisation, if electronic prescribing is adopted, it must comply with Regulation 33’s requirement for an advanced electronic signature (AES).^5,8^

These duties align with global patient-safety guidance: World Health Organization (WHO) documentation standards emphasise that all health records must be legible, clear and free of ambiguous abbreviations to ensure safe, continuous care. World Health Organization’s clinical-documentation guidance stresses legibility as a minimum requirement for effective communication among practitioners and for preventing medication and diagnostic errors. Similarly, the WHO Guide to Good Prescribing warns that unclear handwriting and non-standard shorthand directly contribute to avoidable prescribing errors.^12,13^ The HPCSA’s requirement that records be ‘complete, legible and understandable’ therefore mirrors WHO’s minimum documentation standards and reflects international best practice.^11^

The consequences of illegibility are far-reaching.^4^ In medical negligence cases, courts often rely on contemporaneous clinical notes to assess the standard of care provided. When these notes are unreadable or ambiguous, they fail to support the clinician’s version of events, weakening the defence and increasing the likelihood of adverse findings. Moreover, illegible documentation can delay proceedings, inflate legal costs, and contribute to the growing burden of medico-legal claims against provincial health departments.

## South African legal position on legibility

What follows is a summary of the status quo regarding legibility in South Africa:

- **Prescriptions:** Regulation 33 requires legible print or compliant electronic prescriptions signed with an AES. Illegible or non-AES prescriptions are non-compliant.^8^
- **Clinical notes:** HPCSA Booklet 9 requires records to be ‘complete, legible and understandable’. Persistent non-compliance may amount to unprofessional conduct.^11^
- **Evidence:** Courts frequently treat hospital notes as hearsay unless admitted under statute; clarity affects credibility and weight.^9,10^

The South African Law Reform Commission has identified deficient record-keeping systems as a central driver of the escalating medico-legal crisis, noting that incomplete, inaccurate and missing documentation impede both clinical management and the defence of negligence claims.^5^ Crucially, illegible handwriting forms a core component of poor record-keeping: when clinical notes, prescriptions and medico-legal reports cannot be reliably interpreted by anyone other than the author, they become functionally inaccessible, producing the same downstream harms as missing or inadequate records. This connection is underscored by the HPCSA’s Booklet 9, which explicitly requires records to be complete, accurate and legible, emphasising that illegibility renders documentation non-compliant with professional standards.^9^ Evidence from across the public sector confirms how these deficiencies contribute to the medico-legal burden. Ndebele shows that poor documentation – alongside staff shortages and systemic constraints – drives rising malpractice claims, draining billions of rands that could otherwise fund health services.^1,5^ Kolawole’s national review similarly demonstrates that non-compliant record-keeping, including handwritten notes that are unclear or indecipherable, undermines investigations and litigation, weakening both patient care and the adjudication of negligence cases.^2^ The Medical Protection Society has long warned that illegible notes expose clinicians to avoidable liability and compromise continuity of care by obscuring key clinical information.^3^ In rural and under-resourced settings, where digitisation remains limited and staff turnover is high, these risks are amplified: handwritten records remain the primary source of information, and any ambiguity can result in misinterpreted instructions, incorrect medication, delayed interventions and ultimately preventable harm to patients and substantial financial and reputational consequences for providers.^1,2,3^

Ultimately, illegibility in clinical documentation is not a benign oversight – it is a systemic vulnerability with profound implications for justice, safety and the sustainability of the healthcare system. Addressing this issue requires not only technological investment but also a cultural shift in how documentation is taught, valued and audited within medical practice.^3^

South Africa’s medico-legal environment is especially vulnerable.^11^ In cases involving sexual offences, assault or negligence, medico-legal reports often serve as critical evidence. In such matters, illegible or incomplete documentation can undermine both clinical accuracy and the evidentiary value of the record, exposing practitioners to professional and legal risk. Ensuring that records meet the HPCSA’s documentation standards is therefore essential to maintaining both ethical practice and evidentiary integrity.^11^

Despite the existence of clear HPCSA standards requiring that all clinical records – whether handwritten or electronic – be legible, complete and contemporaneous, many public hospitals and clinics continue to rely heavily on handwritten documentation because of resource constraints, limited digitisation and inconsistent training.^2^ Handwriting itself is not contrary to HPCSA requirements. However, the risk arises when handwriting is difficult to read, in all settings, as this increases the likelihood of clinical misinterpretation and corresponding legal vulnerabilities. These challenges highlight the centrality of legibility as a minimum standard of safe and ethical practice and provide a natural link to the discussion that follows.

## The court in India

While the South African court underscores the importance of legible notes (*Madida obo M v MEC for Health for the Province of KwaZulu-Natal*, para 11)^9,14^ it is mostly silent about the quality of writing of handwritten records, the Indian court addressed the issue explicitly. In the High Court of Punjab & Haryana,^4,6^ the petitioner sought anticipatory bail under Section 438 of the Code of Criminal Procedure (Cr.P.C).^15^ Multiple allegations were made: fraud, impersonation of naval officers, obtaining money under false pretexts for recruitment into the Intelligence Bureau, and allegations of rape. During proceedings, the Court examined the MLR, which was entirely illegible (p. 2, para 2). This illegibility, along with similarly unreadable prescriptions in a related matter (CRM-M-9887-2025), prompted the High Court of Punjab & Haryana (India)^6^ to initiate a broader inquiry into illegible handwriting in medical documentation across public and private hospitals.

The issue before this court was whether a legible medical prescription and diagnosis constitutes an integral part of the Right to Health and therefore forms part of the Fundamental Right to Life under Article 21 of the Constitution of India (p. 15. para 20).^16^

Turning to the legal framework governing medical practice in India, the Punjab & Haryana High Court^6^ rooted its analysis in Article 21,^16^ which recognises the Right to Health as part of the right to life. This constitutional framing parallels South Africa’s own commitment to access to healthcare under section 27 of the Constitution^17^ and reinforces the principle that patients have a right to clear, comprehensible medical information.

The Court further drew on WHO clinical-documentation standards, which require that records be legible and free of ambiguous abbreviations to ensure safe, continuous care – standards that align directly with the HPCSA’s requirement that patient records be ‘complete, legible and understandable’.^12,13^

Professional obligations under the Indian Medical Council and National Medical Commission regulations require doctors to issue legible prescriptions, preferably in capital letters. This requirement is substantively consistent with South Africa’s Regulation 33,^8^ which mandates legible prescriptions and imposes formal validity standards for printed and electronic prescriptions.

The Indian Court also highlighted statutory duties under the *Clinical Establishments Act*,^18^ which require accurate, accessible medical records. This converges with South Africa’s *National Health Act*,^7^ which similarly obliges health establishments to maintain proper, accessible patient records capable of supporting safe clinical care and legal scrutiny.^19^

Importantly, the Indian judgement recognised that illegibility undermines informed consent, obstructs proper investigation, and delays adjudication.^6^ South African evidence law reflects the same concern: courts treat unclear or illegible hospital notes as hearsay unless properly admitted, and poor-quality documentation weakens evidentiary weight and medico-legal defensibility.^9,10,14^

Taken together, the Indian Court’s reasoning does not diverge from South African doctrine. Instead, it reinforces and constitutionalises principles already embedded in South African law: that legibility is integral to accountability, continuity of care, evidentiary integrity and patient dignity.

## Recommendations for South Africa

Given the understanding that handwritten notes with its concomitant risks will be part of the South African Health landscape for the foreseeable future, the health system needs to adopt a multi-pronged approach. The multi-pronged reform strategy must aim to mitigate the systemic risks posed by illegible clinical documentation and strengthen medico-legal accountability. The following recommendations aim to address both the structural and normative dimensions of the problem:

- **Curriculum reform and professional development:** Medical education should integrate formal training in legible documentation, medico-legal literacy, and ethical record-keeping. This includes teaching students the evidentiary role of clinical notes, the legal consequences of poor documentation, and the standards set by the HPCSA.^11^ Continuing professional development (CPD) programmes should reinforce these principles for practising clinicians, with targeted workshops in high-risk disciplines such as emergency medicine, obstetrics and forensic pathology.^3^
- **Digitisation and infrastructure investment:** The rollout of electronic health records (EHRs) and e-prescribing systems must be accelerated, particularly in public hospitals and rural clinics. Government investment in digital infrastructure should be accompanied by training and support to ensure effective implementation.^1,2^ Digitisation not only improves legibility but also enhances data security, continuity of care and auditability – key components of a resilient health system.^2^
- **Policy enforcement and institutional accountability:** Oversight mechanisms must be strengthened to ensure compliance with HPCSA guidelines on record-keeping.^11^ This includes routine audits of patient files, disciplinary action for repeated non-compliance, and the development of institutional policies that prioritise legibility and documentation quality. Hospital management should be held accountable for ensuring that staff are adequately trained and resourced to meet medico-legal standards.^5^
- **Legal recognition and constitutional framing:** Consideration should be given to legislative or judicial recognition of legibility as an essential component of the constitutional right to access healthcare services. This framing would elevate the issue from a matter of professional etiquette to one of legal entitlement, reinforcing the idea that patients have a right to clear, comprehensible, and accurate medical records. Such recognition could also serve as a basis for legal recourse in cases where illegible documentation contributes to harm or injustice.^1^
- **Equity and legibility:** In clinical practice, handwriting that is clear, consistent and easily interpretable is essential to safe and dignified care. Poorly legible notes can impede communication, delay treatment, and disproportionately disadvantage vulnerable patients who rely on accurate, timely information to understand their health status and care plan. Ensuring that handwritten records are readable is therefore not simply a technical requirement but an ethical one: legibility upholds the principles of equity, fairness and respect within healthcare by enabling all patients to benefit from accurate documentation.^3^

## Conclusion

Legibility in clinical documentation is not a peripheral concern; it is fundamental to patient safety, evidentiary integrity and the fair administration of justice.^6,8,11,12,13^ In the South African context – where medico-legal claims continue to escalate, and public healthcare resources are under substantial pressure – clear, accessible records must be understood as both a legal obligation and an ethical imperative.

The recent Indian judgement^6^ underscores this point by framing legibility as intrinsic to the right to health. Its reasoning, although grounded in a different constitutional tradition, aligns closely with South Africa’s existing statutory and professional standards. As South Africa advances towards broader adoption of EHRs and e-prescribing systems, it is vital that principles of clarity, legibility and accountability remain central. Achieving this will require strengthening documentation training within medical curricula, investment in digital infrastructure, and consistent enforcement of medico-legal responsibilities across the health system.

Recognising legibility as a component of the constitutional right to access healthcare offers a coherent framework for reform. It positions clear documentation not as an administrative preference, but as a safeguard against medical error, preventable harm, and systemic inequity. In a multilingual society where patients already face barriers to understanding clinical information, illegible handwriting magnifies these disparities.

Ultimately, this issue demands careful attention. Something as routine as handwriting can determine whether care is safe, whether justice is done, and whether patients’ rights are meaningfully realised. Ensuring legible clinical records is therefore essential to strengthening accountability, improving patient outcomes and upholding dignity within South Africa’s healthcare system.
